# The Effects of Free Heme on Functional and Molecular Changes During *Ex Vivo* Normothermic Machine Perfusion of Human Kidneys

**DOI:** 10.3389/fimmu.2022.849742

**Published:** 2022-05-02

**Authors:** Sarah A. Hosgood, Tegwen R. Elliott, Nina P. Jordan, Michael L. Nicholson

**Affiliations:** Department of Surgery, Addenbrooke’s Hospital, University of Cambridge, Cambridge, United Kingdom

**Keywords:** normothermic perfusion, kidney, free heme, hemolysis, immune response

## Abstract

Normothermic machine perfusion (NMP) is a technique of kidney preservation designed to restore cellular metabolism after cold ischemia. Kidneys are perfused with an oxygenated banked red blood cell (RBC) based solution for 1h at 36°C. During NMP, RBCs can become damaged, releasing free heme into the perfusate. This can act as a damage-associated molecular pattern (DAMP) activating inflammatory signalling pathways. The aim of this study was to measure the levels of free heme during NMP, assess the effect on kidney function and determine any association with inflammatory and stress related gene expression. Levels of free heme were measured in perfusate samples from a series of donation after circulatory death (DCD) kidneys undergoing NMP as part of a randomised controlled trial (RCT). The age of RBCs and levels of free heme were correlated with perfusion parameters. Changes in gene expression were analysed in a series of kidneys declined for transplantation using the NanoString nCounter Organ Transplant Panel and qRT-PCR. Older units of RBCs were associated with higher levels of free heme and levels increased significantly during NMP (Pre 8.56 ± 7.19µM vs 26.29 ± 15.18µM, P<0.0001). There was no association with levels of free heme and perfusion parameters during NMP (P > 0.05). Transcriptional and qPCR analysis demonstrated the upregulation of differentially expressed genes associated with apoptosis (FOS and JUN), inflammatory cytokines (IL-6, SOCS3, ATF3), chemokines (CXCL8, CXCL2, CC3/L1) and oxidative stress (KLF4) after NMP. However, these did not correlate with levels of free heme (P >0.05). A significant amount of free heme can be detected in the perfusate before and after NMP particularly when older units of red cells are used. Although transcriptional analysis demonstrated significant upregulation of genes involved with apoptotic, inflammatory and oxidative pathways these were not associated with high levels of free heme.

## Introduction

Normothermic machine perfusion (NMP) technologies are being trialled in clinical practice to improve early graft function and assess the quality of kidneys for transplantation (1–3). An oxygenated red cell-based solution is circulated through the kidney under near physiological conditions. Cellular function is restored to replenish ATP and minimize the effects of cold ischemia.

NMP can be carried out using a number of different strategies. Experimentally, prolonged durations of NMP have shown benefit in improving early graft function (4). However, in clinical practice shorter durations performed after hypothermic preservation at the recipient center are logistically more practical. Preliminary studies have demonstrated the safety and feasibility of this technique in clinical practice (5) and the results of a RCT in DCD kidneys (1) are expected later this year.

Compatible packed red blood cells (pRBCs) from a local blood bank are used in most NMP circuits as an oxygen carrier. RBCs are also important regulators of vascular tone and nitric oxide bioavailability (6). Although pRBCs can be stored for up to 42 days, over time their condition deteriorates (7). During storage the destruction of the RBC membrane causes the release of haemoglobin and free heme (8, 9). This is further exacerbated during NMP when RBCs come into contact with artificial surfaces and by the perfusion pump (10, 11). Excess free heme is highly toxic and has the ability to induce oxidative stress, inflammation, cellular injury and apoptosis (10, 12). Free heme also binds nitric oxide derived from the endothelium reducing nitric oxide availability to cause vasoconstriction, thrombin formation, fibrin deposition, platelet activation and aggregation that leads to organ dysfunction (10, 12).

The aim of this study was to measure the levels of free heme during NMP and assess the effect on kidney function and to determine any association with inflammatory and stress related gene expression.

## Materials and Methods

Kidneys undergoing 1h NMP from a RCT (ISRCTN15821205) were used to determine the effect of free heme on perfusion parameters (n = 42). The study protocol and trial documents were approved by the NHS Health Research Authority East of England, Cambridge Central Research Committee (15/EE/0356).

Human kidneys rejected for transplantation and offered for research were used to investigate the transcriptional effects of free heme on gene expression and cellular damage (n = 15). Consent for research was obtained by specialist nurses in organ donation (SNODs). The study was approved by the National Research Ethics committee and Research and Development office at the University of Cambridge (NRES: 15/NE/0408).

### Normothermic Machine Perfusion

NMP was carried out for 1 hour after a period of static cold storage as previously described (1). In brief, the NMP was performed using adapted cardiopulmonary bypass technology (Medtronic) and consisted of a centrifugal pump, membrane oxygenator, heat exchanger, venous reservoir and PVC tubing.

The circuit was primed with one unit of compatible packed red blood cells (RBCs) mixed with a priming solution (**Supplementary Table 1**). Supplements were added to support kidney function. In the research kidneys creatinine (1500µmol/L) was added to the perfusate to measure creatinine clearance. Kidneys were flushed with 500ml cooled Ringer’s solution (4°C) prior to being connected to the NMP circuit

The renal artery was cannulated and kidneys were perfused at a pump speed of 1450 RPM. The perfusate temperature was maintained between 35 and 37°C and mean arterial pressure of 75–85mmHg. Renal blood flow (RBF), arterial pressure, temperature and urine output were monitored throughout.

### Quality Assessment Score

During NMP the quality of each transplanted kidney was graded. The NMP quality assessment score was derived from three factors: macroscopic appearance, mean RBF and total urine output. Kidneys with a quality assessment score of 4 or less were considered suitable for transplantation provided there were no other preclusions to transplant (13).

### Perfusate and Urine Analysis

Perfusate samples were collected before and after NMP. Samples were centrifuged at 1600 RPM for 10 minutes at 4°C. Supernatant was removed, flash frozen in liquid nitrogen and stored at –80°C. Urine samples were collected after 1h NMP. Samples were centrifuged at 1600 RPM for 10 minutes at 4°C. Supernatant was removed, flash frozen in liquid nitrogen and stored at –80°C.

### Free Heme

Free heme was measured in the perfusate samples before and after NMP using the Heme Assay Kit (Sigma-Aldrich, St Louis, USA) following manufacturer’s instructions.

### Tissue Samples

In the research kidneys, wedge biopsies of cortex were taken before and after NMP. Biopsies were divided into three for fixation in 10% formalin (CellStor, CellPath, Powys, UK) for paraffin wax embedding, flash frozen in liquid nitrogen and fixed in RNALater Stabilisation Solution (Invitrogen, ThermoFisher, California, USA).

Apoptosis was measured using TUNEL staining on 4µm sections of paraffin fixed tissue. TUNEL staining was carried out following manufacturer’s instructions (abcam, Cambridge, UK). Sections were viewed at ×200 magnification and ten fields of view were imaged. TUNEL positive cells were counted in each field of view and an average number of positive cells was calculated for each sample. Apoptotic cells were identified by dark brown staining.

### Injury Markers

Protein lysates were prepared from tissue samples collected before and after NMP. Tissue (5mg) was homogenised in RIPA lysis buffer (Sigma-Aldrich, St Louis, USA) with protease inhibitor cocktail (Sigma-Aldrich, St Louis, USA) using mechanical homogeniser. Samples were left on ice for 30 mins and then centrifuged for 20 minutes at 4°C 12000 RPM.

The protein lysates were used to measure oxidative stress in the research kidneys using the Protein Carbonyl content assay kit (RayBiotech, Georgia, USA) following manufacturer’s instructions.

Human neutrophil gelatinase-associated lipocalin (NGAL) was measured in the urine at the end of NMP following manufacturer’s instructions (Cohesion Bioscience, London, UK).

### NanoString Analysis

Gene expression profile was performed using the Human Organ Transplant panel from NanoString technologies. Total RNA was isolated from kidney biopsies and 770 genes related to organ transplant pathways were screened according to manufacturer’s instructions. Normalization, differential of expression and pathways analysis were performed within the nSolver Software Advanced Analysis. Normalisation was based on the selection of a panel of housekeeping genes through geNorm pairwise variation statistic. Differential of expression was displayed in a volcano plot with p-value adjusted by the Benjamini–Hochberg false discovery rate correction method. Further analysis was performed within the R software: the heatmap and principal component analysis were plotted with the top 25 and 100 differentially expressed genes respectively.

### Gene Expression

Gene expression of IL-6, TLR4, HO-1, HMGB1, FOS and JUN (ThermoFisher, California, USA) (**Supplementary Table 2**) was quantified using RT qPCR on RNAlater fixed tissue in 15 of the research kidneys. RNAlater fixed tissue was ground in lysis buffer (Qiagen, Maryland, USA) using a micropestle. The samples were homogenised. Chloroform was added and the samples were shaken vigorously and centrifuged for 15 minutes at 12000g and 4°C. The upper phase was collected and RNA extraction was carried out using RNAEasy Mini Kit following manufacturer’s instructions (Quiagen, Maryland, USA). The RNA elution was immediately stored on ice to prevent RNA degradation. RNA concentration and quality was measured using NanoDrop Spectrophotometer ND-1000.

Reverse transcription (RT) was carried out on RNA samples using a High Capacity cDNA Reverse Transcription Kit (Applied Biosystems, Fisher Scientific, California, USA) following manufacturer’s instructions. Briefly, each sample was mixed with the master mix provided and RT was carried out in the thermal cycler (BioRad T100) at 25°C for 10 minutes, 37°C for 120 minutes, 85°C for 5 minutes and 4°C until used in qPCR.

qPCR was carried out on the BioRad CFX RealTIme System. Samples were analysed in triplicate and each experimental well contained SsoAdvanced Universal Probes Supermix (BioRad, Hertfordshire, UK), primer (**Supplementary Table 2**), cDNA and RNAase free water. PCR consisted of 95°C for 10 seconds followed by 60°C for 30 seconds for a total of 40 cycles.

Fold increase of expression was quantified using the delta delta ct method with 18s used as the gene of reference to which samples were normalized. Gene expression was presented as a fold change after NMP compared to baseline biopsies taken prior to NMP.

### Statistical Analysis

Continuous data were tested for normality and the appropriate statistical test carried out. Data were compared using Student’s t-test or Wilcoxon test. Correlations to determine associations between levels of free heme measured in the perfusate before and after NMP and age of pRBC, levels of potassium, lactate, functional perfusion parameters and gene expression were calculated using Pearson’s correlation matrix.

For the transplanted and research kidneys donor demographics and ischemic times were recorded.

To determine any differences between subpopulations of research kidneys dependent on donor type demographics, perfusion parameters, protein expression and gene expression in donation after brain death (DBD) and DCD kidneys were compared.

Values are presented as the mean ± SD for parametric data and median and inter-quartile range (IQR) for non-parametric distributed data. P values of >0.05 were considered significant. Statistical analysis was carried out using Prism statistical software version 9 (GraphPad, California, USA).

## Results

### Clinical Series 

A total of 42 kidneys underwent NMP with pRBCs. Donor demographics and ischemic times are listed in **Table 1**. Levels of free heme and lactate increased significantly during NMP (P<0.0001, 0.0004; **Table 1**) but levels of potassium remained stable (P =0.2795; **Table 1**).

**Table 1:** Donor demographics and perfusion outcomes (n=42) of DCD kidneys that underwent 1h NMP prior to transplantation.

Demographics		N=42	Statistical significance
Donor age (years)		54 ± 14	
Donor gender *% Female*		36	
Donor cause of death *(n)*	*ICH* *Hypoxic brain damage* *Intracranial thrombosis* *Respiratory failure* *Pneumonia* *Cardiac arrest* *Pulmonary embolism*	12 17 3 2 1 6 1	
Terminal creatinine (µmol/L)		78.8 ± 43.5	
Warm ischemic time (minutes)		11.4 ± 2.5	
Cold ischemic time (minutes)		824.2 ± 268.1	
Free heme (uM)	*Pre NMP* *1h NMP*	8.56 ± 7.19 26.29 ± 15.18	<0.0001
Lactate (mmol/L)	*Pre NMP* *1h NMP*	8.26 ± 2.17 10.13 ± 3.63	0.0004
Potassium (mmol/L)	*Pre NMP* *1h NMP*	10.01 ± 2.81 10.53 ± 2.83	0.2795
QAS (n)	*1* *2* *3* *4*	21 16 4 1	
RBF (ml/min/100g)		73.10 ± 20.49	
Urine output (ml)		130.2 ± 104.4	
NGAL (ng/ml)		16.06 ± 13.22	
Oxygen consumption (ml/mg/g)		45.67 ± 17.86	

Data are presented as mean and standard deviation from the mean. Parameters measured pre and post NMP were compared and statistical significance determined (Student’s paired t test).

ICH, intracerebral hemorrhage; NGAL, neutrophil gelatin lipocalin; NMP, normothermic machine perfusion; QAS, quality assessment score; RBF, renal blood flow.

The majority of kidneys were of good quality and had a quality assessment score of 1 and 2 (**Table 1**). The mean RBF was 73.1 ± 20.5ml/min/100g, total urine output 130 ± 104ml and urine NGAL 16.1 ± 13.2ng/ml (**Table 1**).

### Correlation With Perfusion Parameters in the Clinical Kidneys 

Older units of pRBCs were associated with higher levels of free heme, higher levels of potassium and lactate before and after 1h NMP (P <0.05). Baseline levels of free heme were associated with significantly higher levels of baseline potassium (P = 0.044), baseline lactate (P = 0.001), 1h lactate (P = 0.001) and 1h free heme (P <0.0001).

**Figure 1:**
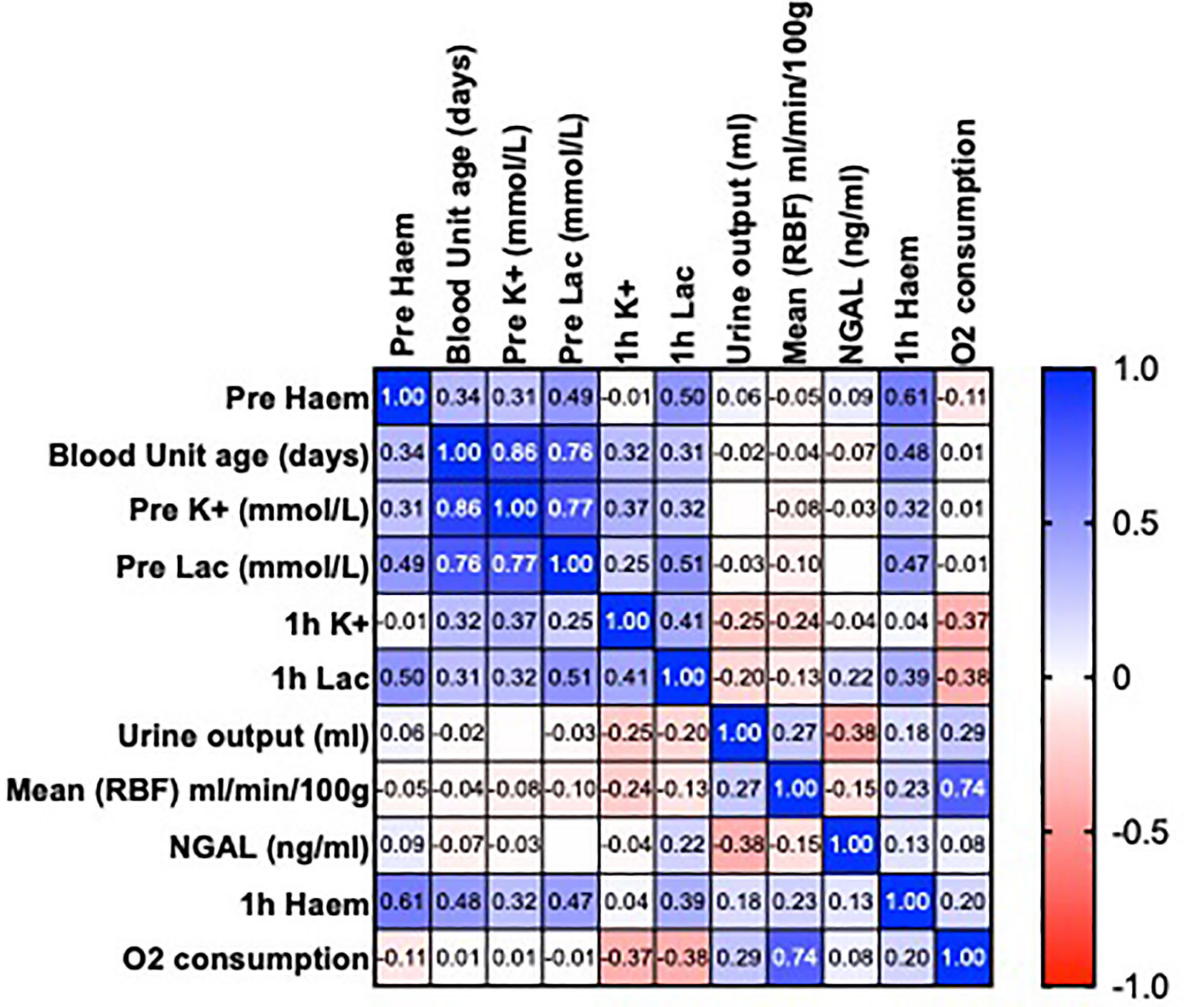
Heatmap showing Pearson’s correlation matrix to examine the associations of the age of packed red blood cells and levels of free heme on perfusion parameters before and after 1h of normothermic machine perfusion (NMP). Values are R.

There was no association with the age of pRBCs or levels of free heme and functional parameters (urine output, RBF, oxygen consumption and NGAL; P >0.05) (**Figure 1**).

### Research Kidneys

**Figure 2:**
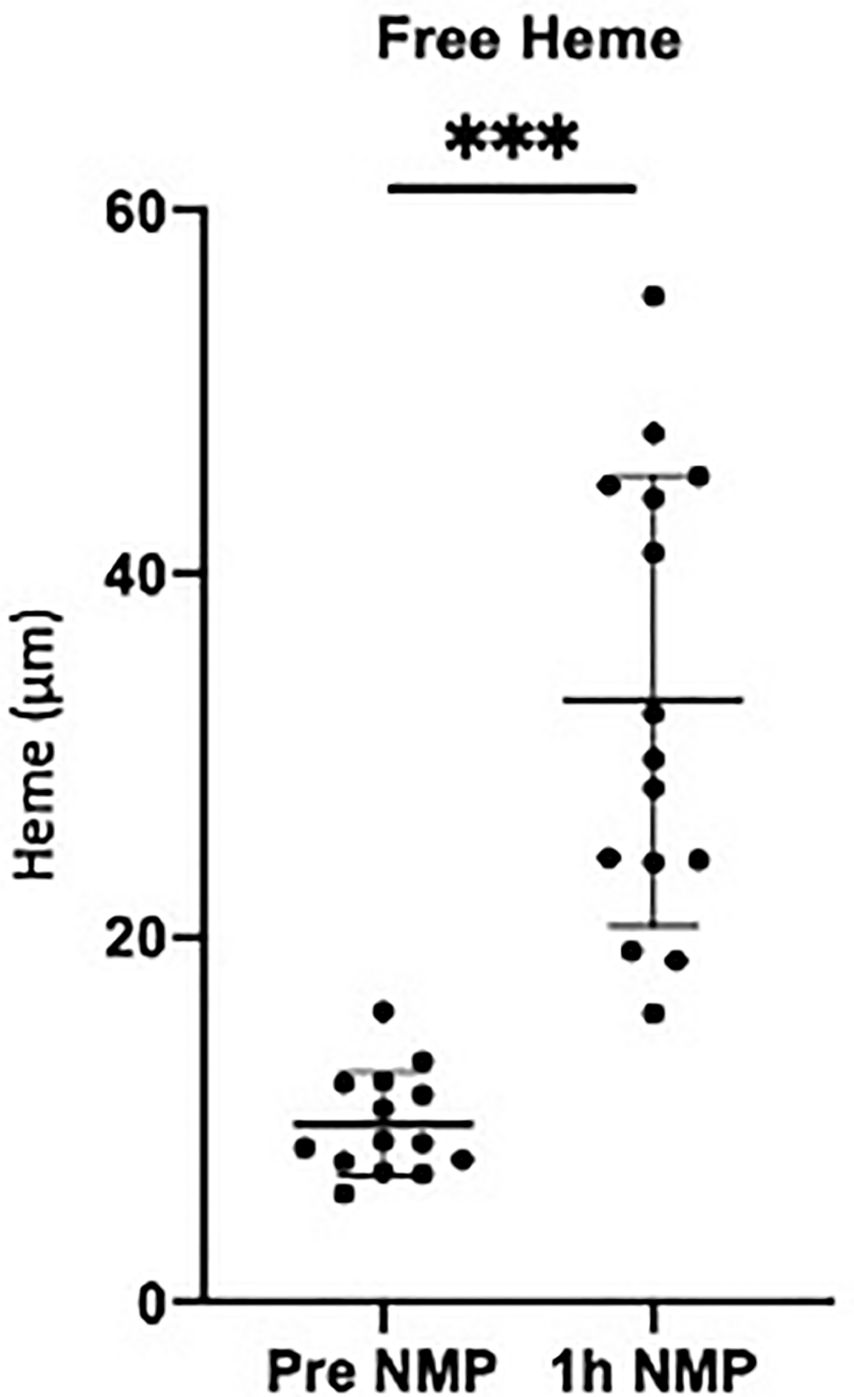
Levels of free heme measured in the perfusate pretransplant and post 1h NMP in a series of human kidneys that were rejected for transplanted and offered for research. Data presented as mean (SD). ***P < 0.0003

The donor characteristics and ischemic times for the research kidneys (10 DBD and 5 DCD) are detailed in **Table 2**. The average cold ischemic time before NMP was 1395 ± 498 minutes (**Table 2**). Mean urine output was 83 ± 55ml, NGAL 3.1 ± 4.2ng/ml and RBF 68.9 ± 31.2 ml/min/100g. The average age of pRBCs was 25 ± 8 days (**Table 2**). Levels of free heme in the perfusate increased significantly during NMP (P = 0.001; **Figure 2**).

**Table 2:** Donor demographics and ischemic times for research kidneys (n=15) that underwent 1h NMP.

Donation type	Donor age (Years)	Left/Right Kidney	WIT (mins)	CIT (mins)	Reason for decline	Cause of death	Urine output (ml)	Urine NGAL (ng/ml)	Mean RBF (ml/min/100g)	Age of pRBCs (days)
DBD	64	Right	na	2051	Enlarged lymph nodes	Cardiac arrest	35	–	73.3	21
DBD	60	Left	na	653	Suspected malignancy	ICH	120	14.01	102.7	23
DBD	72	Right	na	1380	Past medical history	ICH	92	16.32	69.5	33
DCD	53	Left	17	1674	Cut ureter	ICH	92	39.46	78.2	19
DBD	75	Left	na	2034	Remuzzi score	ICH	164	10.16	104.8	14
DBD	45	Right	na	1545	Suspected malignancy	ICH	116	15.22	82.0	37
DCD	68	Right	10	1773	Remuzzi score	ICH	120	33.70	92.2	35
DBD	58	Left	na	1248	Dissection renal artery	Hypoxic brain damage	14	10.27	53.2	17
DBD	60	Left	na	1483	Damage to ureter	Trauma - unknown cause	30	46.61	40.9	35
DCD	74	Right	10	1327	Remuzzi score	ICH	145	21.11	128.5	29
DBD	56	Left	na	1154	Suspected malignancy	Septicaemia	10	17.31	32.3	21
DBD	76	Right	na	536	Diseased aortic patch	ICH	28	24.19	31.6	37
DBD	75	Left	na	711	Atheroma renal artery	ICH	25	8.41	28.2	18
DCD	59	Right	18	1140	Damage to renal artery	ICH	164	9.58	81.3	22
DCD	67	Right	9	1647	Damage to renal vein	ICH	90	16.24	34.0	19

CIT, cold ischemic time; DBD, Donation after brain death; DCD, donation after circulatory death; ICH, intracerebral hemorrhage; NMP, normothermic machine perfusion; WIT, warm ischemic time. na, not applicable.

#### Transcriptional Gene Expression 

NanoString nCounter Organ Transplant Panel was used to examine the transcriptional changes in gene expression in four human DBD research kidneys that underwent 1h NMP.

**Figure 3:**
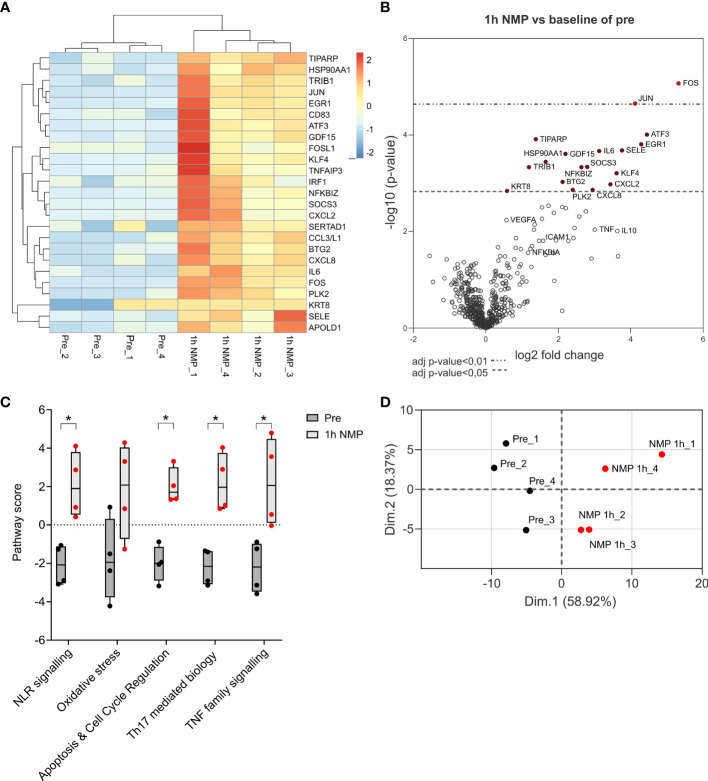
Nanostring nCounter Organ Transplant Panel analysis of 4 cortex samples of kidneys comparing transcriptional analysis of differential expressed genes after 1h NMP compared to baseline pre samples. Total RNA was isolated and processed. **(A)** Heatmap of the top 25 significantly differentially expressed genes. **(B)** Volcano plot represents differential gene expression after 1h NMP. Difference in gene expression level with p-value adjusted <0.01 or <0.05 are marked by red dots. **(C)** Top 5 pathways up-regulated after NMP. Statistical significance was calculated by Mann Whitney test (*, p<0.05). **(D)** Principal component analysis on the top 100 differentially expressed genes.

Seventeen differentially expressed genes were significantly upregulated after NMP. These included genes associated with apoptosis (FOS and JUN), inflammatory cytokines (IL-6, SOCS3, ATF3), chemokines (CXCL8, CXCL2, CC3/L1) and oxidative stress (KLF4). Several genes associated with anti-inflammatory properties (IL-10) and endothelial cell recovery (VEGF) were also upregulated though did not reach statistical significance (**Figures 3A, B**). Pathway analysis showed that the top five significantly upregulated pathways after 1h NMP were involved in nod like receptor (NLR) signalling, oxidative stress, apoptosis and cell cycle regulation, Th17 mediated biology and TNF family signalling (**Figure 3C**). Principal component analysis demonstrated a distinct separation of the pre- and post-1h NMP samples (**Figure 3D**)

#### Quantification (qPCR) 

PCR analysis of the 15 research kidneys showed a significant increase in expression of FOS (P < 0.0001), median fold increase 59 (3-364), IL-6 (P = 0.0001), median fold increase 11 (3-81), JUN (P = 0.0001), median fold increase 11 (1-146) and TLR4 (P = 0.0353), median fold increase 1.4 (0.3-16.2). There was no significant fold change in HO-1 (P = 0.2293) and HMGB1 (P = 0.446; **Table 3**).

**Table 3:** Expression of genes in tissue measured using qPCR.

Gene name	Fold change (median (IQR))	Significance (p value)
FOS	59.03 (21.91–98.13)	<0.0001
IL-6	11.02 (6.32–41.46)	0.0001
JUN	11.24 (5.71–17.07)	0.0001
TLR4	1.44 (1.00–3.04)	0.0353
HO-1	1.15 (0.65–3.04)	0.2293
HMGB1	1.03 (0.49–3.07)	0.4457

Measured as median fold change (IQR) post NMP compared to samples taken prior to NMP. Wilcoxon test for significance (p < 0.05)

#### Correlations With Gene Expression and Perfusion Parameters in the Research Kidneys 

**Figure 4:**
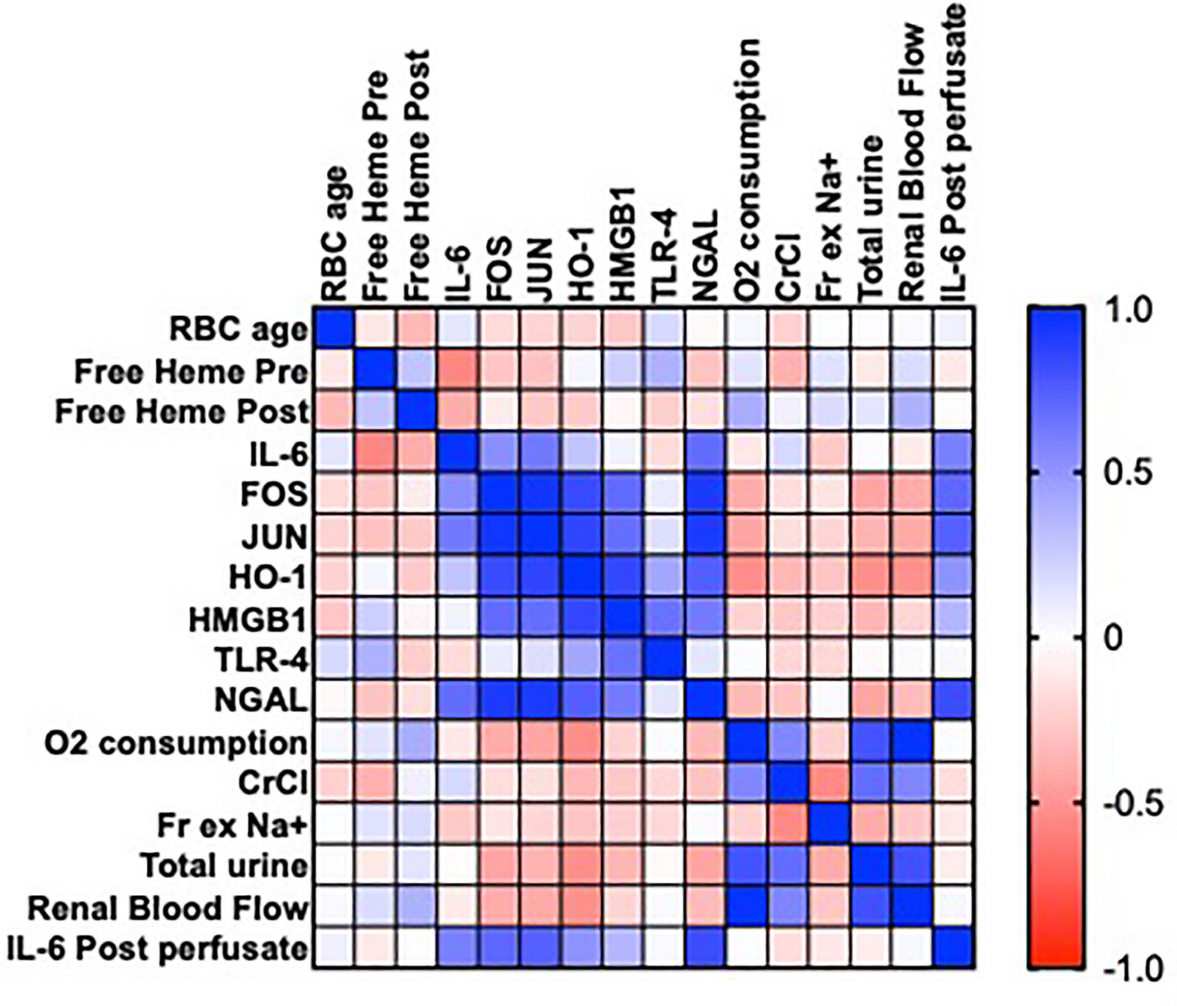
Heatmap showing Pearson’s correlation matrix in a series of 15 human kidneys rejected for transplantation to examine the associations of the age of packed red blood cells (pRBC) and levels of free heme on gene expression (IL-6, FOS, JUN, HO-1, HMGB1 and TLR-4) after NMP, measures of renal function (oxygen consumption, creatinine clearance [CrCl], fractional excretion of sodium [Fr Ex Na+], total urine output, renal blood flow), levels of urinary neutrophil gelatinase associated lipocalin (NGAL) and perfusate levels of IL-6 post NMP. Values are R.

pRBC age and levels of free heme pre and post NMP did not correlate with changes in gene expression (IL-6, FOS, JUN, HO-1, HMGBI, TLR4 P >0.05; **Figure 4**) in the discard series. There were associations between the expression of IL-6 and FOS and JUN (P = 0.048, 0.015, respectively; **Figure 4**), HO-1, FOS, JUN and HMGB1 (P <0.05; **Figure 4**) and HMGB1, FOS, JUN and TLR4 (P<0.05; **Figure 4**)

pRBC age and levels of free heme pre and post NMP also did not correlate with each other or with perfusate levels of IL-6 or levels of functional parameters (P>0.05; **Figure 4**). There was a significant positive correlation between levels of IL-6 and urinary NGAL (r=0.824, ***P<0.0003; **Figure 4**).

#### Oxidative Stress and Apoptosis

**Figure 5:**
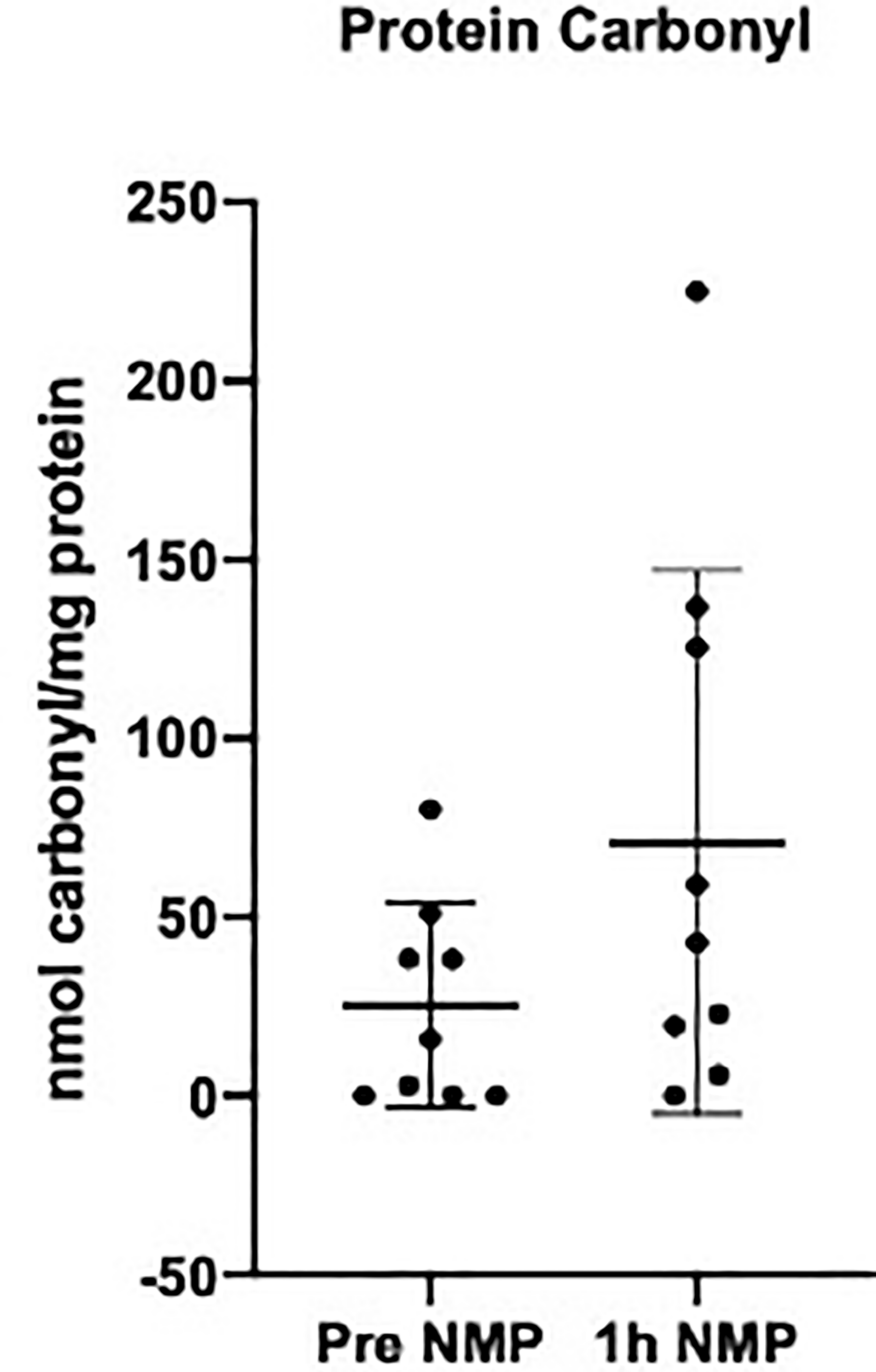
Levels of protein carbonyl quantifying oxidative stress measured in the perfusate pretransplant and post 1h NMP in a series of human kidneys that were rejected for transplant and offered for research. Data presented as mean (SD).

There was a numerical increase in levels of protein carbonyl after NMP; however, this did not reach statistical significance (P = 0.115) (**Figure 5**).

**Figure 6:**
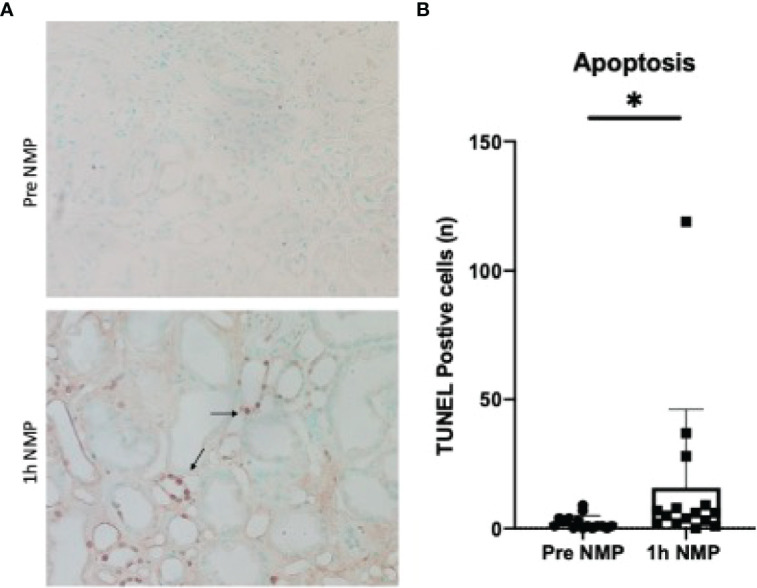
**(A)** TUNEL staining of apoptotic cells in pre and post cortical biopsies. Positive cells stained brown (highlighted by arrows). **(B)** Quantification of TUNEL positive staining. Average number of cells per 10 fields of view. Data is presented as mean (SD).*P = 0.027

The number of apoptotic cells significantly increased during NMP compared to baseline samples (P = 0.027). The majority of positive staining was found in the tubular epithelial cells (**Figures 6A, B**).

### Comparison of DCD and DBD Research Kidneys

A subanalysis was carried out to determine any differences between the DCD and DBD kidneys. There was no significant difference in the mean donor age (DBD 66 ± 10y, DCD 62 ± 9y; P = 0.241) and the cold ischemic time was matched between groups (DBD 1512 ± 267 min, DCD 1360 ± 573 min; P = 0.588).

Baseline and 1h NMP levels of free heme were similar between DCD and DBD kidneys (baseline P = 0.699, 1h NMP P = 0.254).

DBD kidneys had a significantly greater fold change in IL-6 gene expression compared to the DCD kidneys after 1h NMP (DCD 6.08 (4.09-14.83), DBD 29.51 (8.41-52.25); P = 0.029). There were no significant differences between groups in the five other target genes (FOS, JUN, HO-1, HMGB1, TLR4; P > 0.05).

## Discussion

The NMP system uses adapted clinical grade pediatric cardiopulmonary bypass technology designed to minimize hemolysis. However, factors such as the heat generated by the centrifugal pump, shear stress, negative pressures, turbulent flow and gravity assisted venous drainage contribute to hemolysis and release of free heme. In cardiac bypass procedures excess levels of heme are associated with organ damage and increased mortality (11, 14). The use of banked RBCs for NMP is problematic in that during storage the deformability and surface charge of RBCs decreases and fragility and aggregability increases (15, 16). This makes them more susceptible to damage during NMP. During storage there is also an accumulation of lipids and RBC derived microparticles or macrovesicles that act as damage-associated molecular patterns (DAMPs), which can stimulate the immune system and production of reactive oxygen species (17, 18). In the clinical series, we found that older units of pRBCs had significantly higher levels of free heme at baseline and levels increased further during NMP. Furthermore, older units of pRBCs also had higher levels of potassium and lactate before and after NMP. This is presumably due to leakage from the fragile RBCs during storage. Perfusate levels of lactate increased significantly during perfusion but it is difficult to determine whether this was due to further damage of the red cells or increased lactate production due to inadequate tissue oxygenation (19). Interestingly, in the discard kidney series the age of pRBCs was not associated with increased levels of free heme but levels increased significantly during NMP. This may be due to the small sample size and variation within this group. Nonetheless, in both the transplanted and discarded kidneys no association was found between the levels of free heme and renal perfusion parameters including oxygen consumption which suggests no detrimental effect and adequate tissue oxygenation. There was also no evidence of significant oxidative damage at the level of protein expression or in the number of apoptotic cells within this short NMP timeframe.

To determine the molecular effects of heme, changes in transcriptional gene expression in a number of the discarded kidneys were assessed. There was significant upregulation of cellular stress related genes that are consistent with ischemic injury. Genes upregulated after NMP included heat shock proteins, oxygen free radicals (KLF4), hypoxia and inflammatory genes (IL-6, SOCS3, ATF3) and chemokines (CXCL8, CXCL2, CC3/L1). FOS and JUN gene transcription were amongst the top five significantly upregulated differentially expressed genes. They regulate activator protein 1, a prominent transcription factor involved with regulating cellular fate involving the mitogen-activated protein kinases (MAPKs) pathways (ERK, p38 and JNK). Together with the NLR pathway, the MAPK pathway is involved in the downstream signalling of the innate immune response and pro-inflammatory cytokine production with activation of NF-κB/NLRP3 inflammasome (20).

To quantify the findings from the transcriptional data, a number of genes were investigated using qRT-PCR. In agreement with the NanoString transcriptomic data, there was a significant upregulation of IL-6, FOS and JUN. We also measured HMGB1, a nuclear protein that in situations of stress and ischemia, translocates from the nucleus to the cytoplasm and is excreted into the extracellular space to act as a DAMP (21, 22). HMGB1 has proinflammatory interactions with downstream receptors such as toll like receptor-4 (TLR4). The HMGB1/TLR4 pathway is well recognized in the pathophysiological process of renal ischemic injury and acute kidney injury activating NF-κB mainly through the MyD88 pathway (23). In this study we found a significant upregulation of TLR4 after NMP but not HMGB1. Dexamethasone is a glucocorticoid receptor that suppress NF-κB and MAPK-ERK activation and the subsequent translocation of HMGB1 (24). It is added to the perfusate during NMP as an anti-inflammatory agent and may therefore account for our findings.

There was no association in levels of free heme and gene expression in the series of discarded kidneys. However, based on the known effects of free heme we cannot dismiss the possibility of its contribution to the stimulation of these inflammatory and oxidative stress pathways. With such a short perfusion time, we were unable to quantify the protein expression of these pathways. As a DAMP, free heme influences the expression of many genes and, in the NMP environment in the absence of plasma proteins, free heme can interact with TLR4 to upregulate inflammatory mediators (23, 25, 26). Pathway analysis showed upregulation of NLR, oxidative stress, apoptosis, Th17 and TNF family signalling, all of which can be stimulated by free heme (27). Surprisingly, hemeoxygenase-1 (HO-1), an important enzyme rapidly stimulated to counteract the detrimental effects of free heme by catalyzing heme to carbon monoxide, ferrous iron and biliverdin (28), was not upregulated during NMP. Again, this may be due to the addition of dexamethasone during NMP which can suppress its expression (29).

The upregulation of inflammatory mediators during NMP has been associated with prolonged early graft dysfunction (30) and therefore efforts should be made to improve the NMP environment, including reducing levels of free heme, this may be particularly important for longer periods of NMP. Younger units of pRBCs would be advantageous but free heme, potassium, lactate and microparticles can be removed by washing the RBCs before use. However, this may affect the quality of the RBCs increasing their fragility and susceptibility to mechanical stress during NMP (8). Dilution of the RBCs with plasma or albumin can reduce inflammation and oxidative damage by binding to free heme (29).

The addition of an oxygen carrier during NMP is considered essential for adequate oxygen delivery to the tissues. Venema L et al, found that the addition of RBCs during more prolonged periods of NMP of porcine kidneys resulted in higher levels of oxygen consumption and function compared to conditions without RBCs (19). Artificial oxygen carriers such as hemopure are an alternative to RBCs and are being trialled in kidney NMP (31, 32). Subnormothermic temperatures or the combination of subnormothermic temperature and artificial oxygen carriers have recently been shown to reduce inflammation and pulmonary vascular resistance during ex vivo lung perfusion (EVLP) (33) and should be explored in kidney NMP.

Another strategy is to use an oxygenated acellular solution to rewarm kidneys in a controlled manner. Zlatev H et al. recently published the results of six extended criteria donor kidneys demonstrating the safety and feasible of the technique (34). Nonetheless, this has not be trialled for longer periods of perfusion.

This is the first study to examine the effects of free heme during kidney NMP. It involved two populations of kidneys: to determine the effects of free heme on kidney function during NMP a series of DCD kidneys included in a RCT were used; to determine the effect of free heme at a molecular level a series of DBD and DCD kidneys rejected for transplantation and offered for research were used. Although the research kidneys had longer cold ischemic times and were from DBD and DCD donors, levels of free heme and functional parameters during NMP were similar to the RCT kidneys. The population of DCD research kidneys were further disadvantaged with more of the DCD kidneys being perfused with older units of pRBCs. The small sample size limits any conclusion on the effect of free heme on DCD compared to DBD kidneys in this study but previous analysis of transcriptional gene expression after NMP has found results to be similar between DBD and DCD kidneys (30).

In conclusion, high levels of free heme are found in units of stored RBCs and increase significantly during 1h of NMP. There was no direct association between levels of free heme, kidney function or the upregulation of inflammatory and stress related genes during perfusion. However, this may have consequences for longer periods of perfusion and levels of hemolysis should be minimized during NMP.

## Data Availability Statement

The data presented in the study are deposited in the GEO repository, accession number (GSE197304) https://www.ncbi.nlm.nih.gov/geo/query/acc.cgi?acc=GSE197304)

## Ethics Statement

The studies involving human participants were reviewed and approved by East of England, Cambridge Central Research Committee (15/EE/0356), National Research Ethics committee and Research and Development office at the University of Cambridge (NRES: 15/NE/0408). The patients/participants provided their written informed consent to participate in this study.

## Author Contributions

SH conceived the research idea, planned and conducted the experiments, analysed the results and co-wrote the manuscript; TE conducted the experiments, analysed the results and co-wrote the manuscript; NJ conducted the experiments, analysed the results and revised the manuscript; MN conceived the research idea and revised the manuscript. All authors contributed to the article and approved the submitted version.

## Funding

The research was funded Kidney Research UK and by the National Institute for Health Research Blood and Transplant Research Unit (NIHR BTRU) in Organ Donation and Transplantation at the University of Cambridge in collaboration with Newcastle University and in partnership with NHS Blood and Transplant (NHSBT) Grant RG75628.

## Author Disclaimer

The views expressed are those of the authors and not necessarily those of the NIHR, the Department of Health and Social Care or NHSBT.

## Conflict of Interest

The authors declare that the research was conducted in the absence of any commercial or financial relationships that could be construed as a potential conflict of interest.

## Publisher’s Note

All claims expressed in this article are solely those of the authors and do not necessarily represent those of their affiliated organizations, or those of the publisher, the editors and the reviewers. Any product that may be evaluated in this article, or claim that may be made by its manufacturer, is not guaranteed or endorsed by the publisher.

## Abbreviations

ATF3, activating transcription factor 3; ATP, adenosine triphosphate; CC3/L1, C-C motif chemokine ligand 3 like 1; CXCL8, C-X-C motif chemokine ligand 8; CXCL2, C-X-C motif chemokine ligand 2; DAMP, damage-associated molecular pattern; DBD, donation after brain death; DCD, donation after circulatory death; ERK, extracellular signal-regulated kinase; FOS, Fos proto-oncogene; HMGB1, high mobility group box 1; HO-1, hemeoxygenase 1; IL-6, interleukin 6; IL-10, interleukin 10; JNK, c-Jun N-terminal kinases; JUN, Jun proto-oncogene; KLF4, Kruppel Like Factor 4; MAPKs, mitogen-activated protein kinases; NF-κB, nuclear factor kappa B; NGAL, neutrophil gelatinase-associated lipocalin; NLR, nod like receptor; NLRP3, NLR family pyrin domain containing 3; NMP, normothermic machine perfusion; pRBCs, packed red blood cells; RBCs, red blood cells; RBF, renal blood flow; RCT, randomised controlled trial; RT, reverse transcription; SOCS3, suppressor of cytokine signalling 3; TLR4, toll-like receptor 4; TNF, tumor necrosis factor; VEGF, vascular endothelial growth factor.

## References

[1] Hosgood SA Saeb-Parsy K Wilson C Callaghan C Collett D Nicholson ML. Protocol of a Randomised Controlled, Open-Label Trial of Ex Vivo Normothermic Perfusion Versus Static Cold Storage in Donation After Circulatory Death Renal Transplantation. BMJ Open (2017) 7(1):e012237. doi: 10.1136/bmjopen-2016-012237 PMC5278243 28115329

[2] PROlonged Ex-vivo Normothermic Machine PERfusion for Kidney Regeneration (2020). Available at: https://clinicaltrials.gov/ct2/show/NCT04693325.

[3] GL MD. Normothermic Machine Perfusion (NMP) Compared to Static Cold Storage (SCS) in Donation After Brain Death (DBD) Kidney Transplantation; a Prospective Multicenter Randomized Controlled Trial (NMP-DBD) . Available at: https://clinicaltrials.gov/ct2/show/NCT05031052.

[4] Urbanellis P Hamar M Kaths JM Kollmann D Linares I Mazilescu L. Normothermic Ex-Vivo Kidney Perfusion Improves Early DCD Graft Function Compared to Hypothermic Machine Perfusion and Static Cold Storage. Transplantation (2019) 104(5):947–55. doi: 10.1097/TP.0000000000003066 31815900

[5] Nicholson ML Hosgood SA. Renal Transplantation After Ex Vivo Normothermic Perfusion: The First Clinical Study. Am J Transpl (2013) 13(5):1246–52. doi: 10.1111/ajt.12179 23433047

[6] Helms CC Gladwin MT Kim-Shapiro DB. Erythrocytes and Vascular Function: Oxygen and Nitric Oxide. Front Physiol (2018) 9:125. doi: 10.3389/fphys.2018.00125 29520238 PMC5826969

[7] Flegel WA Natanson C Klein HG. Does Prolonged Storage of Red Blood Cells Cause Harm? Br J Haematol (2014) 165(1):3–16. doi: 10.1111/bjh.12747 24460532 PMC5515544

[8] Cardigan R New HV Tinegate H Thomas S. Washed Red Cells: Theory and Practice. Vox Sang (2020) 115(8):606–16. doi: 10.1111/vox.12971 32633823

[9] García-Roa M Del Carmen Vicente-Ayuso M Bobes AM Pedraza AC González-Fernández A Martín MP. Red Blood Cell Storage Time and Transfusion: Current Practice, Concerns and Future Perspectives. Blood Transfus Trasfus Sangue (2017) 15(3):222–31. doi: 10.2450/2017.0345-16 PMC5448828 28518049

[10] Zakkar M Guida G Suleiman M-S Angelini GD. Cardiopulmonary Bypass and Oxidative Stress. Oxid Med Cell Longev (2015) 2015:189863. doi: 10.1155/2015/189863 25722792 PMC4334937

[11] Vercaemst L. Hemolysis in Cardiac Surgery Patients Undergoing Cardiopulmonary Bypass: A Review in Search of a Treatment Algorithm. J Extra Corpor Technol (2008) 40(4):257–67. PMC4680715 19192755

[12] Chiabrando D Mercurio S Tolosano E. Heme and Erythropoieis: More Than a Structural Role. Haematologica (2014) 99(6):973–83. doi: 10.3324/haematol.2013.091991 PMC4040894 24881043

[13] Hosgood SA Thompson E Moore T Wilson CH Nicholson ML. Normothermic Machine Perfusion for the Assessment and Transplantation of Declined Human Kidneys From Donation After Circulatory Death Donors. Br J Surg (2018) 105(4):388–94. doi: 10.1002/bjs.10733 PMC5887977 29210064

[14] Cholette JM Pietropaoli AP Henrichs KF Alfieris GM Powers KS Gensini F. Elevated Free Hemoglobin and Decreased Haptoglobin Levels are Associated With Adverse Clinical Outcomes, Unfavorable Physiologic Measures, and Altered Inflammatory Markers in Pediatric Cardiac Surgery Patients. Transfusion (Paris) (2018) 58(7):1631–9. doi: 10.1111/trf.14601 PMC6105435 29603246

[15] Himbert S Qadri SM Sheffield WP Schubert P D’Alessandro A Rheinstädter MC. Blood Bank Storage of Red Blood Cells Increases RBC Cytoplasmic Membrane Order and Bending Rigidity. PloS One (2021) 16(11):e0259267. doi: 10.1371/journal.pone.0259267 34767588 PMC8589153

[16] Ichikawa J Koshino I Arashiki N Nakamura F Komori M. Storage-Related Changes in Autologous Whole Blood and Irradiated Allogeneic Red Blood Cells and Their Ex Vivo Effects on Deformability, Indices, and Density of Circulating Erythrocytes in Patients Undergoing Cardiac Surgery With Cardiopulmonary Bypass. J Cardiothorac Vasc Anesth. (2022) 36(3):855–861. doi: 10.1053/j.jvca.2021.06.016 34253445

[17] Hoehn RS Jernigan PL Chang AL Edwards MJ Pritts TA. Molecular Mechanisms of Erythrocyte Aging. Biol Chem (2015) 396(6–7):621–31. doi: 10.1515/hsz-2014-0292 PMC5673117 25803075

[18] Tzounakas VL Georgatzakou HT Kriebardis AG Voulgaridou AI Stamoulis KE Foudoulaki-Paparizos LE. Donor Variation Effect on Red Blood Cell Storage Lesion: A Multivariable, Yet Consistent, Story. Transfusion (Paris) (2016) 56(6):1274–86. doi: 10.1111/trf.13582 27028307

[19] Venema LH van Leeuwen LL Posma RA van Goor H Ploeg RJ Hannaert P. Impact of Red Blood Cells on Function and Metabolism of Porcine Deceased Donor Kidneys During Normothermic Machine Perfusion. (2021). doi: 10.1097/TP.0000000000003940 PMC9128616 34456268

[20] Hao JL Li YF Li RS. A Novel Mechanism of NALP3 Inducing Ischemia Reperfusion Injury by Activating MAPK Pathway in Acute Renal Failure. Med Hypotheses (2013) 80(4):463–5. doi: 10.1016/j.mehy.2012.12.041 23399110

[21] Florim GMS Caldas HC Gonçalves NN Bueno GOBE Baptista MASF Fernandes-Charpiot IMM. Activation of HMGB1–TLR4 Pathway and Inflammasome Contribute to Enhanced Inflammatory Response in Extended Criteria and Kidneys With KDPI ≥85%. Transplantation (2020) 104(4):724–30. doi: 10.1097/tp.0000000000003048 31764760

[22] Chen C-B Liu L-S Zhou J Wang X-P Han M Jiao X-Y. Up-Regulation of HMGB1 Exacerbates Renal Ischemia-Reperfusion Injury by Stimulating Inflammatory and Immune Responses Through the TLR4 Signaling Pathway in Mice. Cell Physiol Biochem (2017) 41(6):2447–60. doi: 10.1159/000475914 28472797

[23] Zhao Z Hu Z Zeng R Yao Y. HMGB1 in Kidney Diseases. Life Sci (2020) 259:118203. doi: 10.1016/j.lfs.2020.118203 32781069

[24] Zhang J Xia J Zhang Y Xiao F Wang J Gao H. Hmgb1-TLR4 Signaling Participates in Renal Ischemia Reperfusion Injury and Could be Attenuated by Dexamethasone-Mediated Inhibition of the ERK/NF-κb Pathway. Am J Transl Res (2016) 8(10):4054–67. PMC5095301 27829992

[25] Habib R. Multifaceted Roles of Toll-like Receptors in Acute Kidney Injury. Heliyon (2021) 7(3):e06441. doi: 10.1016/j.heliyon.2021.e06441 33732942 PMC7944035

[26] Janciauskiene S Vijayan V Immenschuh S. Tlr4 Signaling by Heme and the Role of Heme-Binding Blood Proteins. Front Immunol (2020) 11:1964. doi: 10.3389/fimmu.2020.01964 32983129 PMC7481328

[27] Vallelian F Schaer CA Deuel JW Ingoglia G Humar R Buehler PW. Revisiting the Putative Role of Heme as a Trigger of Inflammation. Pharmacol Res Perspect (2018) 6(2):e00392. doi: 10.1002/prp2.392 29610666 PMC5878102

[28] Chiabrando D Vinchi F Fiorito V Mercurio S Tolosano E. Heme in Pathophysiology: A Matter of Scavenging, Metabolism and Trafficking Across Cell Membranes. Front Pharmacol (2014) 5:61. doi: 10.3389/fphar.2014.00061 24782769 PMC3986552

[29] Deramaudt TB da Silva JL Remy P Kappas A Abraham NG. Negative Regulation of Human Heme Oxygenase in Microvessel Endothelial Cells by Dexamethasone. Proc Soc Exp Biol Med Soc Exp Biol Med N Y N (1999) 222(2):185–93. doi: 10.1046/j.1525-1373.1999.d01-130.x 10564544

[30] Ferdinand JR Hosgood SA Moore T Ferro A Ward CJ Castro-Dopico T. Cytokine Absorption During Human Kidney Perfusion Reduces Delayed Graft Function-Associated Inflammatory Gene Signature. Am J Transplant Off J Am Soc Transplant Am Soc Transpl Surg (2020). doi: 10.1111/ajt.16371 PMC8246774 33098231

[31] Aburawi MM Fontan FM Karimian N Eymard C Cronin S Pendexter C. Synthetic Hemoglobin-Based Oxygen Carriers are an Acceptable Alternative for Packed Red Blood Cells in Normothermic Kidney Perfusion. Am J Transpl (2019) 19(10):2814–24. doi: 10.1111/ajt.15375 PMC6763345 30938927

[32] Bhattacharjee RN Patel SVB Sun Q Jiang L Richard-Mohamed M Ruthirakanthan A. Renal Protection Against Ischemia Reperfusion Injury: Hemoglobin-Based Oxygen Carrier-201 Versus Blood as an Oxygen Carrier in Ex Vivo Subnormothermic Machine Perfusion. Transplantation (2020) 104(3):482–9. doi: 10.1097/TP.0000000000002967 31568396

[33] Arni S Necati C Maeyashiki T Opitz I Inci I. Perfluorocarbon-Based Oxygen Carriers and Subnormothermic Lung Machine Perfusion Decrease Production of Pro-Inflammatory Mediators. Cells (2021) 10(9):2249. doi: 10.3390/cells10092249 34571898 PMC8466246

[34] Zlatev H von Horn C Kaths M Paul A Minor T. Clinical Use of Controlled Oxygenated Rewarming of Kidney Grafts Prior to Transplantation by Ex Vivo Machine Perfusion. A Pilot Study. Eur J Clin Invest (2022) 52(2):e13691. doi: 10.1111/eci.13691 34747502

