# Supplementary material for: The Effects of Free Heme on Functional and Molecular Changes During Ex Vivo Normothermic Machine Perfusion of Human Kidneys
Source: Front Immunol. 2022 May 2;13:849742. doi: 10.3389/fimmu.2022.849742 (PMC9108696; doi:10.3389/fimmu.2022.849742)
Supplement: Supplementary file 1 [file DataSheet_1.docx]

**Supplementary information**

| Perfusate component | Volume | Infusion Rate |
| --- | --- | --- |
| Packed red blood cells | 1 Unit | - |
| Ringer’s Solution | 250ml | - |
| Mannitol 10% | 15ml | - |
| Dexamethasone (3.3mg/ml) | 2ml | - |
| Heparin sodium | 3000IU | - |
| Sodium Bicarbonate 8.4% | 27ml | - |
| Synthamin 17 (with Insulin 100IU, Sodium bicarbonate 8.4% 15ml, multivitamins 5ml) | - | 20ml/h |
| 0.9% sodium chloride with prostacyclin | - | 4ml/h |
| Glucose 5% | - | 4ml/h |

*Table 1 Perfusate components used during 1h NMP*

| **Gene Symbol** | **Gene Name** | **Assay ID** | **Manufacturer** |
| --- | --- | --- | --- |
| PPIA | peptidylprolyl isomerase A | Hs04194521_s1 | ThermoFisher |
| 18s | Eukaryotic 18s rRNA | Hs99999901_s1 | ThermoFisher |
| IL-6 | Interleukin 6 | Hs00174131_m1 | ThermoFisher |
| TLR-4 | Toll Like Receptor-4 | Hs00152939_m1 | ThermoFisher |
| HO-1 | Heme Oxygenase 1 | Hs01110250_m1 | ThermoFisher |
| HMGB1 | High mobility group box protein 1 | Hs01923466_g1 | ThermoFisher |
| FOS | c-FOS | Hs99999140_m1 | ThermoFisher |
| JUN | c-JUN | Hs01103582_s1 | ThermoFisher |

*Table 2 TaqMan probes used in qPCR.*
